# Single-nucleus transcriptomics illuminates sex differences during murine *Escherichia coli* pyelonephritis

**DOI:** 10.1038/s42003-026-09946-8

**Published:** 2026-03-31

**Authors:** Teri N. Hreha, Abigail L. Manson, Christina A. Collins, Haojia Wu, Christophe Georgescu, Benjamin D. Humphreys, Ashlee M. Earl, David A. Hunstad

**Affiliations:** 1 Department of Pediatrics, Washington University School of Medicine, St. Louis, MO USA; 2 Infectious Disease and Microbiome Program, Broad Institute of MIT and Harvard, Cambridge, MA USA; 3 Department of Medicine, Washington University School of Medicine, St. Louis, MO USA; 4 Genomics Platform, Broad Institute of MIT and Harvard, Cambridge, MA USA; 5 Department of Molecular Microbiology, Washington University School of Medicine, St. Louis, MO USA; 6 Present Address: University of Southern Denmark, Odense, Denmark

**Keywords:** Cellular microbiology, Pathogens

## Abstract

There are profound sex differences in the prevalence and outcomes of urinary tract infections (UTI). While females comprise the majority of infections, males exhibit higher morbidity and mortality with upper-tract UTI. Correspondingly, preclinical modeling has demonstrated that male and androgen-exposed female mice are highly susceptible to severe high-titer pyelonephritis, a phenotype observed in < 20% of females. Here we subject kidneys from female, male, and androgen-exposed female C3H/HeN mice with pyelonephritis and PBS-exposed control mice to single-nucleus RNA sequencing, creating (to our knowledge) the first whole-kidney single-nucleus transcriptomic dataset reflecting an infected state, comprising 248,483 nuclei. We differentiate healthy cell populations from those affected during UTI and show sex-discrepant responses that extend to kidney cell types beyond those directly interacting with bacteria. Female responses to UTI comprise a more limited range of cell types exhibiting significant upregulation of genes within KEGG pathways and pro-inflammatory transcription factor regulons. Meanwhile, males evidence predisposition to injury pathways even with control (saline) inoculation and responded to UTI with less intensity but across more cell types than females. In total, these data illuminate sex-discrepant transcriptional responses and outcomes in renal infection and enable detailed dissection of these responses at the cellular and molecular level.

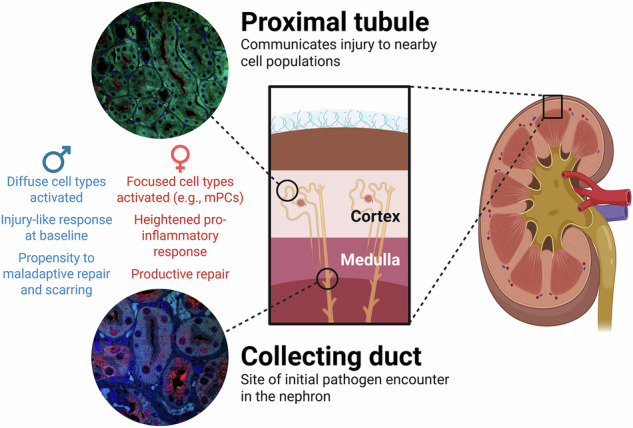

## Abstract (web summary)

Single-nucleus transcriptomics sheds light on cellular pathways underlying sex differences in renal tubular response and repair during kidney infection.

## Introduction

Urinary tract infections (UTIs) affect millions of people worldwide, resulting in millions of dollars spent in hospital visits and treatments^1^. Over 75% of UTIs are caused by uropathogenic *Escherichia coli* (UPEC)^1^, infecting the bladder (cystitis) and often ascending to the kidneys (pyelonephritis). Approximately 50% of women will experience a UTI in their lifetime, and approximately 25% of those women experience recurrent infections^2^. Although UTI in males is less common, elderly men experience UTI at rates similar to those in women, and among infants less than 6 months of age, UTI incidence in boys outpaces that in girls^3^. Further, upper-tract UTIs in men carry increased morbidity and mortality^3,4^.

When UPEC is inoculated into the bladders of female C3H/HeN mice, a background exhibiting vesicoureteral reflux (VUR), 70–80% of these females resolve infection within 7 days after inoculation, while 20–30% experience chronic high-titer infection in both the bladder and kidneys^5^. In contrast, virtually all male and most androgen-exposed female C3H/HeN mice develop persistent high-titer cystitis and pyelonephritis after primary inoculation, in an androgen receptor-dependent manner^6–8^. Most of these mice exhibit renal abscesses, characterized by abundant neutrophil infiltration and leaving renal scars^6–9^. Such abscesses are regional, and the kidneys display zones of both healthy and injured tissue^7^. This distribution complicates studies of the renal response to UTI, especially at early time points, as whole-kidney analyses tend to be insensitive to changes occurring only near foci of infection.

Extensive work has been published using single-nucleus RNA sequencing (snRNAseq) in models of non-infectious acute kidney injury (AKI)^10–13^, enabling a deeper understanding of the renal response to injury, including cellular pathways associated with successful and maladaptive repair. Furthermore, early studies indicate sex-discrepant transcriptional responses to noninfectious AKI^14^, and males are more sensitive to injury in the unilateral ureteral obstruction (UUO) model^15^. Here, we profiled sex-specific host responses to renal infection. We studied kidneys from UPEC-infected female, male, and androgen-exposed female C3H/HeN mice at 5 days post infection (dpi), along with PBS-inoculated control mice, to better understand the effect of host sex on development of pyelonephritis. We chose the C3H/HeN host because kidney infection is robust and sex differences in outcomes are substantial (as detailed above). In addition, the high-grade VUR in these mice models a condition highly associated with pyelonephritis in children^16^ but does not itself cause reflux nephropathy^17^. The time point was selected because bacterial loads are comparable in males and females at this juncture but begin to diverge thereafter^7,8^. As murine pyelonephritis affects regions of the kidney unequally, we utilized the snRNAseq data to distinguish healthy cells from those affected by the experimental UTI. Analysis of sex differences in transcriptional programs in the collecting duct and proximal tubule revealed that females muster a more focused response to infection, with marked upregulation of differentially expressed genes (DEGs) involved in pro-inflammatory transcription factor regulons, and KEGG pathway enrichment particularly in medullary principal cells and injured proximal tubules. Males, on the other hand, exhibited a more diffused response to UTI, with upregulation of DEGs involved in pro-inflammatory pathways across many cell types, reducing delineation between healthy and affected cells. These data indicate that host sex drives divergent responses to UTI across the nephron and illuminate how females better control infection in this model while males are inclined toward chronic infection and renal scarring.

## Results

### Whole-kidney single-nucleus RNA sequencing during active bacterial infection

**Fig. 1:**
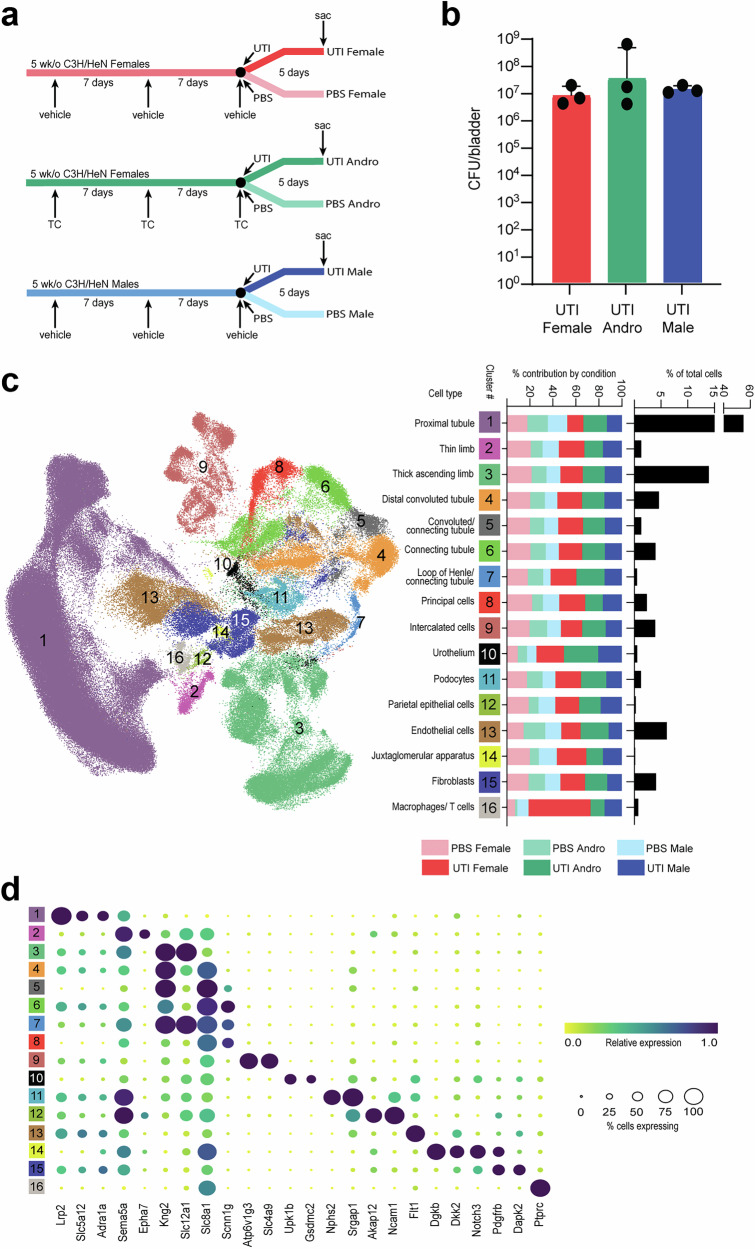
Experimental design and cell clustering. **a** Schematic of experimental design and conditions in this study. Inoculations are indicated with arrows, including TC (testosterone cypionate); vehicle (cottonseed oil); UTI (UPEC strain UTI89 in PBS); or PBS. **b** Bladder titers of the UTI mice selected for kidney single-nucleus RNA sequencing; *p* = 0.83 by Kruskal-Wallis test. Mean ± SD shown. **c** Cluster designations and UMAP diagram for the 16 kidney cell types observed. Bar plots to the right of each cluster number indicate the proportional contribution to each cluster by each experimental condition (with red, green, and blue coloring as in (**a**)); the rightmost column indicates the percent contribution of each cluster to the total number of cells in the dataset. **d** Marker genes identifying the 16 kidney cell types. Circle color indicates relative expression for each gene, while circle size indicates the percent of cells expressing this gene.

snRNAseq was performed on 18 C3H/HeN mice, three from each of six conditions. In the androgen-exposed (Andro) group, female C3H/HeN mice were injected with testosterone cypionate (TC) prior to initiation of UTI^8^, while age-matched female and male C3H/HeN mice were injected with cottonseed oil (vehicle). Mice at 7 weeks of age were inoculated intravesically with UPEC (UTI) or PBS (control) and sacrificed 5 days later (Fig. 1a). Three mice from each UTI group were selected for their high and comparable bladder bacterial loads (Fig. 1b) and visible renal abscesses at the time of sacrifice (Supplementary Fig. 1); three mice from each control (PBS) group were confirmed to have sterile bladders at sacrifice. Nuclei were isolated^18,19^ and RNA sequencing completed. Quality control and filtering of sequencing data included the use of CellBender^20^ to remove ambient RNA contamination, Scrublet^21^ to remove doublets, and Harmony data integration^22^ to correct for batch effects. We obtained sequencing data for a total of 248,483 high-quality single nuclei, representing 41,413 ± 6005 cells per condition, with counts ranging from 4895 to 18,106 cells per mouse. Cells were distributed across 16 expected kidney cell types, defined in part by marker gene expression, as in earlier reports^10,11^ (Fig. 1c, d), and were distributed relatively evenly across conditions, except that the UTI conditions contributed disproportionally to the Urothelium and Macrophage/T cell clusters (Fig. 1c). Recovered immune cells comprised only macrophages and T cells, lacking neutrophils (as previously reported with single-nucleus isolation from kidney, due to their irregular nuclear shapes^10,11^). Of note, one female mouse with UTI contributed disproportionately to this cluster.

### Sex heavily influences transcription at the whole-kidney level

**Fig. 2:**
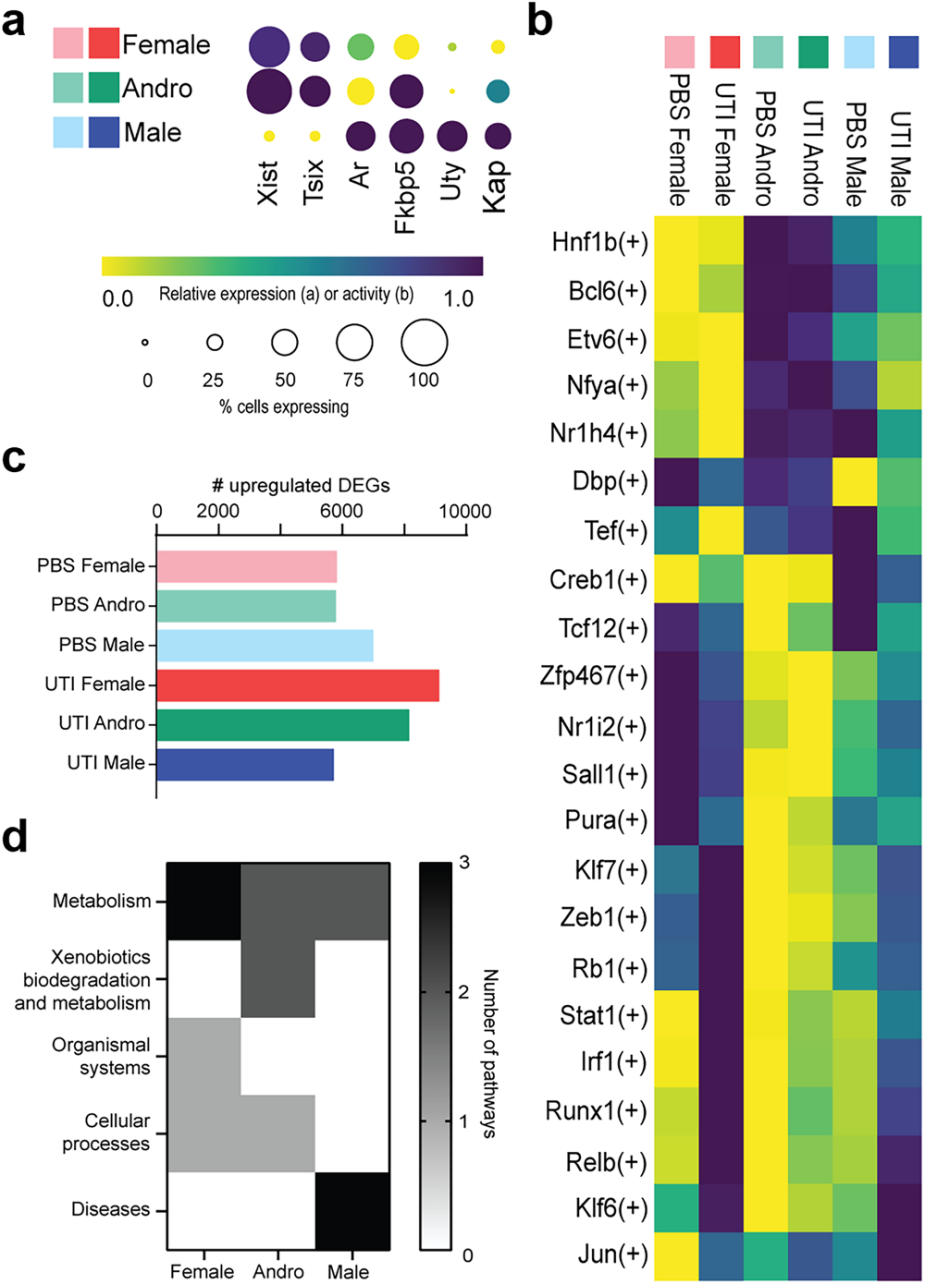
Influence of sex and exogenous testosterone on the kidney. **a** Expression of sex-linked genes in female (red), Andro (green), and male mice (blue). Circle color indicates relative expression for each gene, while circle size indicates the percent of cells expressing this gene. **b** Relative activity of the most differentially active transcription factor regulons in each condition, based on comparisons of outputs from pySCENIC. The superset of the five most highly upregulated regulons during UTI in each condition are plotted. **c** The number of upregulated DEGs (*z*-score > 2.0) in each condition compared to all other conditions. **d** The five most enriched KEGG pathways, based on the 200 most upregulated DEGs in female, Andro, and male mice are categorized by parent family.

To first verify that we could observe expected trends related to sex, we analyzed bulk transcriptional patterns of sex-linked and androgen-responsive genes. As expected, males exclusively expressed the Y-linked transcript *Uty* and exhibited the highest expression of androgen receptor (*Ar*) and the androgen-dependent transcripts *Fkbp5* and *Kap* (Fig. 2a). Furthermore, transcriptional analysis in androgen-exposed female (Andro) mice revealed high expression of X-inactivation genes *Xist* and *Tsix*, similar to that in females, while expression of androgen-responsive genes *Fkbp5* and *Kap* approximated that in males. Andro mice exhibited the lowest expression of *Ar*, reflecting downregulation in response to exogenous testosterone treatment^23^ (Fig. 2a).

To investigate overall trends in expression related to sex, we identified the most differentially active transcription factor (TF) regulons in each condition using pySCENIC^24^, which performs gene regulatory network reconstruction and assesses the activity of regulons in specific cell types. This analysis showed that UTI activated the *Klf6* regulon in both male and female mice, which regulates epithelial-mesenchymal transition and podocyte survival in the kidney^25^ (Fig. 2b and Supplementary Data 1^26^). TF regulons responsible for cellular maintenance, such as *Nr1i2*, *Sal1*, and *Pura*, were highly active in PBS-inoculated females, while several pro-inflammatory regulons, such as those of *Stat1*, *Runx1*, and *Jun*, were highly activated during UTI (compared to PBS conditions).

Interestingly, some of these inflammatory regulons were already modestly active in PBS-inoculated males, indicating that male sex may predispose the kidney toward inflammatory responses to even non-infectious perturbation. Furthermore, during UTI, activity of pro-inflammatory TF regulons such as *Stat1* and *Runx1* was upregulated less strongly in males than in females, in agreement with other reports that female mice exhibit a more robust acute inflammatory response to UTI^6,27^. Aligned with this observation, UTI females exhibited the most upregulated differentially expressed genes (DEGs; *z*-score > 2.0) and the fewest downregulated DEGs (*z*-score < −2.0) compared to all other conditions (Fig. 2c and Supplementary Fig. 2a). PBS males had more upregulated DEGs than UTI males, perhaps reflecting a transcriptional response to inoculation, while UTI males had the most downregulated DEGs overall. Further, the five most enriched KEGG pathways defined by the 200 most upregulated DEGs in female mice (both PBS and UTI conditions) compared to either male or Andro mice reflected metabolism, organismal systems, and cellular processes, while male mice were enriched in disease pathways. This difference highlights the overriding effect exerted by sex on transcriptional regulation in the kidney, regardless of UTI status (Fig. 2d and Supplementary Data 2^26^).

Meanwhile, KEGG pathway analysis in Andro mice indicated that the experimental androgen exposure exerted outsized influence on gene expression in these mice. The most active regulons in all Andro mice, including the male-biased TF regulons *Bcl6*, *Nr1h4*, and *Hnf1b*^28,29^, were similar in both PBS or UTI exposure and were distinct from those most enriched in both male and female UTI (Fig. 2b and Supplementary Data 1^26^). These regulons were among the least active in female mice (Supplementary Fig. 2b) and though some were also expressed in male mice, it was to a much lesser extent. Moreover, Andro mice had significant enrichment of DEGs involved in KEGG pathways related to xenobiotics biodegradation and metabolism, and hormone and drug processing (Fig. 2d and Supplementary Data 2^26^). This domination of transcriptional responses in Andro mice by hormone-processing pathways permeated our downstream analyses. Attempts to exclude these hormone-responsive signatures from the analyses were further precluded by discrepant ambient and mitochondrial RNA signals in the PBS-inoculated Andro group compared to all other groups. As a result, we included the Andro groups in cell-clustering determinations but elected to exclude the Andro groups from our subsequent analyses of the transcriptional effects of UTI, focusing on only the female and male groups.

### Identification of cells affected by UTI

**Fig. 3:**
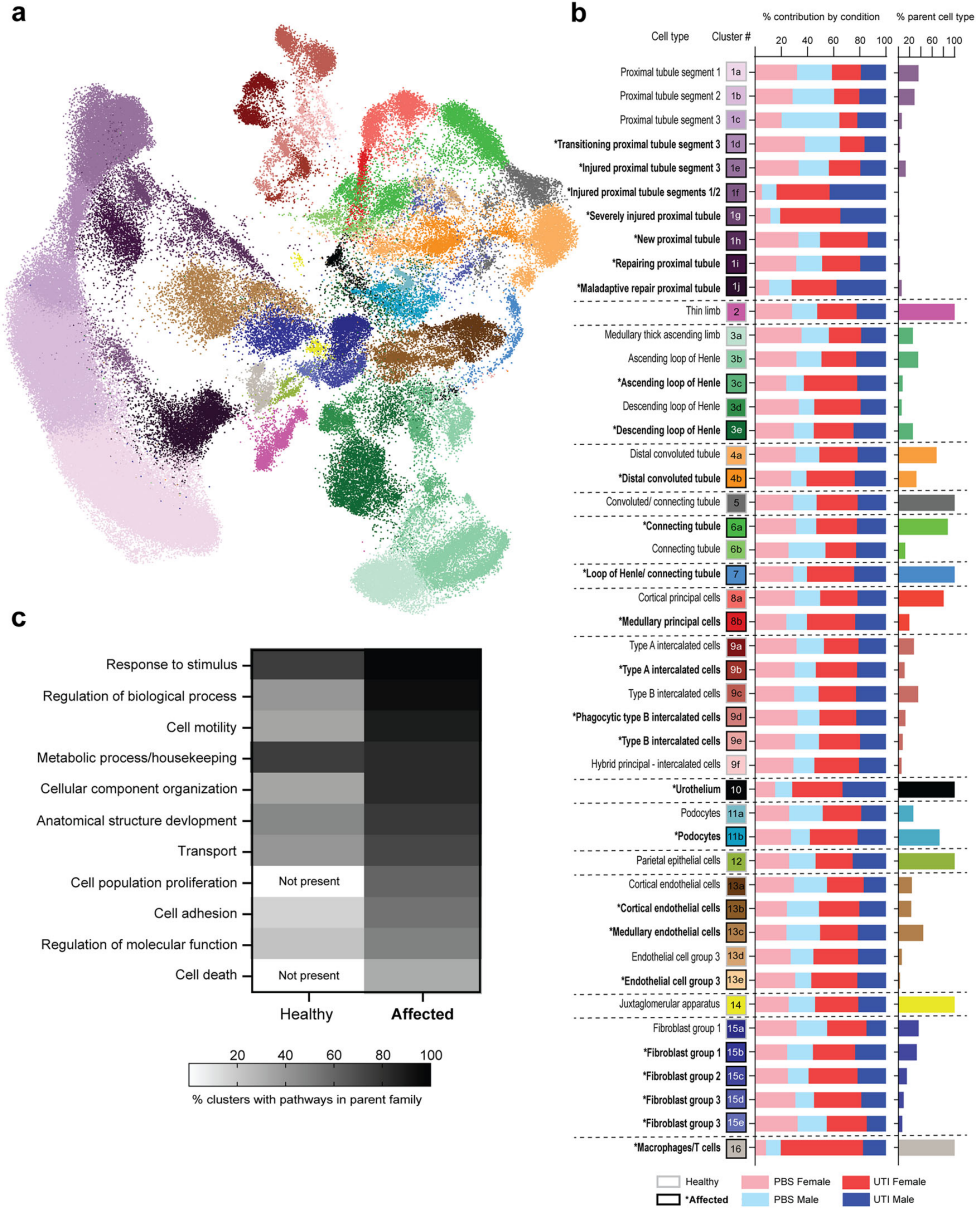
Distinguishment of healthy and affected clusters. **a** UMAP of kidney cell types now further subdivided by cell type specificity as well as “healthy” or “affected” status. **b** Cluster identification detailing the 46 clusters in the UMAP. In the left column, healthy cluster numbers have a gray bounding box, while affected clusters are indicated with an asterisk, bold labeling, and a black bounding box. In the middle column, the proportional contribution to each cluster by each experimental condition is indicated. Each cluster’s contribution to their parent cell type is shown in the rightmost column, colored by their parental cell type (from the 16-cluster scheme in Fig. 1). **c** The most significantly enriched GO terms (FDR < 0.05), derived from the 200 most upregulated DEGs uniquely present in at least three healthy or injured clusters, are plotted as the percent of the total number of healthy or affected clusters with GO terms present in each of the represented parent families.

Because acute and chronic pyelonephritis in C3H/HeN mice is local and regional^7^ (not homogenous across the kidney as is seen in noninfectious injury models), it was important to leverage the snRNAseq dataset to distinguish ***healthy*** (apparently unaffected) cells from those ***affected*** by UTI or by the experimental perturbation of PBS inoculation. During the cluster identification process, we created a set of 46 clusters, identifying kidney cell subtypes aligned with prior studies^10–13,30–36^ and determining which of these cell types were actively responding to UTI (Fig. 3a, b and Supplementary Fig. 3). In the proximal tubule (PT), cell clusters (Fig. 3b, clusters 1a-j) were matched to those described previously in experimental AKI^10^, with the addition of a new transitioning cluster (cluster 1 d), which could not be defined as strictly healthy or affected in our dataset. Cells reflecting injured PT segments 1/2, severely injured PT, and PT undergoing maladaptive repair^10^ (clusters 1 f, g, j) were observed predominantly in mice with UTI and were evenly distributed between females and males (Fig. 3b). For all other non-PT cell types (excluding Macrophage/T cell cluster 16, which were inherently classified as affected), we identified healthy and affected subclusters from the original 98-cluster Leiden. After cell-type identification, healthy versus affected status was determined from the Gene Ontology (GO) terms significantly enriched (FDR < 0.05) among the 200 most upregulated DEGs in each cluster compared to all others. Affected clusters were defined by the presence of GO terms involving regulation of cell death, migration or proliferation, immune cell activation or modulation or activation of, or response to, signaling cascades. Affected clusters in the resulting 46-cluster resolution exhibited enrichment of GO terms such as response to stimulus, regulation of biological process, cell motility and transport, while the upregulated DEGs in healthy clusters predominantly reflected metabolic processes and housekeeping pathways (Fig. 3c). Affected clusters had many more enriched GO terms than healthy clusters (99 vs. 28, respectively; Supplementary Data 3^26^). Importantly, differences in GO term enrichment between healthy and affected cells were analogous to those reported in cells undergoing successful versus maladaptive repair during noninfectious AKI^10^, suggesting overlap in repair processes with diverse injuries. The specific pathways contained within each parent family are listed in Supplementary Data 3^26^. PBS-inoculated mice had cells present in every subcluster, and many affected clusters were similarly contributed to by UTI and PBS mice (Fig. 3b). This observation may reflect a baseline injury-like response due to the vesicoureteral reflux inherent to C3H/HeN mice, or a response to (sterile) inoculation itself.

### Sex differences in the collecting duct response to UTI

As ascending UPEC are known to interact directly with the collecting duct (CD) 5 dpi, forming kidney bacterial communities within the lumen^7,37^, we examined in detail the effects of sex and UTI on CD cell subtypes. The eight CD subclusters (Fig. 3b) included principal cells (PCs; clusters 8a, b), intercalated cells (ICs; comprising IC-A [clusters 9a, b] and IC-B cells [clusters 9c-e]), and hybrid PC-IC subtypes (cluster 9 f). Among PCs, only medullary (m) but not cortical (c) PCs could be classified as affected (Fig. 3b). While the phagocytic capacity of IC-A cells has been reported previously^31^, we identified a population of IC-B cells expressing phagocytic markers including *Dab2*, *Lrp2*, and *Cubn* (cluster 9d; Supplementary Fig. 4a), indicating that these cells may also be able to phagocytose UPEC.

**Fig. 4:**
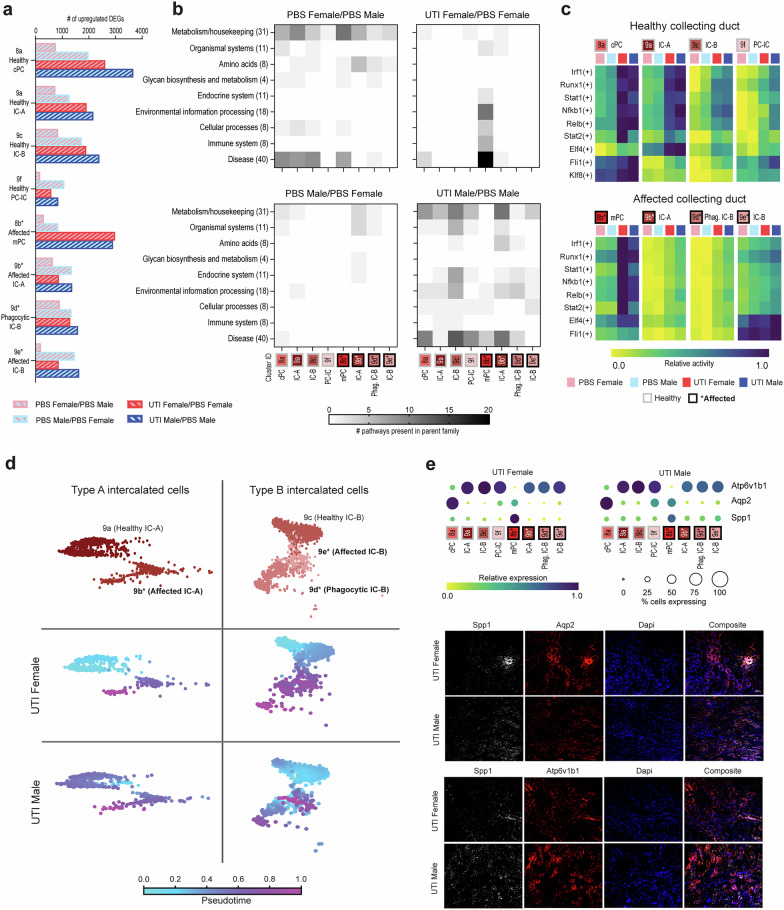
Sex differences in the collecting duct during UTI. **a** The number of upregulated DEGs (*z*-score > 2.0) in each CD subtype, shown for each pairwise comparison as indicated. **b** Heatmaps depicting the number of KEGG pathways within each parent family that were significantly enriched (FDR < 0.05) in the 200 DEGs most highly upregulated in the first condition compared to the second condition. **c** Activity for the most differentially active transcription factor regulons in each CD cluster during UTI. The upper and lower heatmaps show healthy and affected cell types, respectively. For each row, the superset of the five most differentially active regulons during UTI in each cell type are plotted. **d** Pseudotime analysis of type A (left) and type B (right) intercalated cells during UTI. The top row shows the data colored by cell type; the bottom two rows show pseudotime calculations rooted at each healthy cluster, with fuchsia representing cells that are most different from the root. **e** Relative expression of *Spp1*, *Aqp2*, and *Atp6v1b1* in the CD of UTI female and male mice (top), with corresponding IF microscopy images (bottom) showing Spp1 staining (white) in both Aqp2^+^ PCs (top, red) and Atp6v1b1+ ICs (bottom, red) along with Dapi nuclear staining (blue); scale bars, 50 μm. cPC, cortical principal cells; mPC, medullary principal cells; IC-A, type A intercalated cells; IC-B, type B intercalated cells; PC-IC, hybrid principal-intercalated cells; Phag., phagocytic.

We first compared CD cell types in PBS-exposed female and male mice to examine baseline sex differences. PBS males had more significantly upregulated DEGs compared to PBS females in every CD cluster (Fig. 4a). While the 200 most upregulated DEGs in PBS females (i.e., comparatively downregulated in PBS males) reflected KEGG pathways related to metabolism, amino acids, and disease, the most upregulated DEGs in PBS males comprised few enriched KEGG pathways (Fig. 4b, with specific pathways listed in Supplementary Data 4^26^). Meanwhile, by pySCENIC analysis, healthy IC-A and IC-B clusters in PBS males had increased activity in inflammatory TF regulons (e.g., *Runx1*, *Irf1*, *Stat1*, *Nfkb1*) compared to PBS females (Fig. 4c and Supplementary Data 5^26^), indicating that males may be predisposed to injury responses related to VUR or to sterile inoculation.

We then compared CD gene expression in UTI males or females to PBS mice of the same sex. In both males and females, DEG upregulation related to UTI was most notable in principal cells. UTI males had more significantly upregulated DEGs than UTI females throughout the CD, other than in mPCs (cluster 8b; Fig. 4a). Further, among KEGG pathways enriched in the 200 most upregulated DEGs during UTI in females, most were present in mPCs (Fig. 4b, upper right panel). These pathways included several involved in infection response, including environmental information processing, cellular processes, immune system and disease (Fig. 4b; specific pathways in Supplementary Data 4^26^). Interestingly, pathways in these families were downregulated during female UTI (compared to PBS females) in injured IC-As (cluster 9b; Supplementary Fig. 4b), further indicating that mPCs are a primary driver of the infection response in the female CD. In males, UTI provoked a broader response across all CD cell types (Fig. 4b, lower right panel); healthy and affected clusters exhibited similar patterns of KEGG pathway enrichment, whether in pathways involved in cellular homeostasis or in those reflecting cell stress or infection response.

Further, the most active TF regulons in the CD during UTI, based on pySCENIC analysis, also reflected a more dramatic response in affected mPCs (cluster 8b) in females. UTI males had comparatively less activity than UTI females in regulons corresponding to proinflammatory TF regulons such as *Irf1*, *Stat1*, and *Nfkb1*, except in affected IC-A and IC-Bs (clusters 9b and 9e; Fig. 4c and Supplementary Data 5^26^). Downregulated TF regulons are shown in Supplementary Fig. 4c.

We next analyzed sex differences in the intercalated cell clusters of infected mice by pseudotime analysis, which enables representation of progression through a biological process (here infection-related injury) from transcriptomic data^38^. Analyses were rooted in the healthy IC-A and IC-B clusters and performed separately for female and male UTI conditions. Among IC-As (Fig. 4d, left), affected cells were more clearly differentiated from healthy cells along this injury trajectory in females, compared with males. A similar sex difference was evident in IC-Bs; while the trajectory was clear in females, males evidenced less demarcation between healthy and affected cells, or between these clusters and phagocytic IC-Bs (Fig. 4d, right). Taken together, the above set of analyses indicate that CD cell populations respond distinctly to PBS inoculation and UTI depending on sex, even in the context of equivalent bacterial loads.

Of additional note, genes within the KEGG pathway for extracellular matrix-receptor interaction (contained within the environmental information processing family) were significantly upregulated during male UTI in all but mPC, healthy IC-A and PC-IC clusters (clusters 8b, 9a, and 9f) but were expressed only in mPCs during female UTI (Supplementary Data 4^26^). One of the genes in this pathway, *Spp1*, is implicated in renal fibrosis by promoting Smad2/3 phosphorylation and promoting myofibroblast activation^39^, and overexpression of *Spp1* increased renal cell apoptosis during AKI^40^. Indeed, UTI male mice exhibited increased *Spp1* expression throughout the CD compared to UTI females, and immunofluorescence microscopy showed correspondingly more Spp1 staining of the CD in UTI males, with increased colocalization with both Aqp2^+^ PCs and Atp6v1b1^+^ ICs, while Spp1 staining in UTI female mice was more localized to only a few tubules (Fig. 4e). These data suggest that increased *Spp1* expression in the infected male kidney may contribute to the increased prevalence of renal scarring seen in these mice^7,9^.

### Sex differences in the proximal tubule response to UTI

Since proximal tubule (PT) injury has been studied extensively in noninfectious AKI^10–13^, we investigated how sex influences transcriptional activity in PT in the absence and presence of UTI. As noted earlier, cells from both female and male mice with UTI were overrepresented in injured PT segments 1/2 (cluster 1f), severely injured PT (cluster 1g) and maladaptive repair PT (cluster 1j) (Fig. 3b), indicating that UTI elicits injury responses in the PT even if UPEC have not definitively reached this segment of the tubule.

**Fig. 5:**
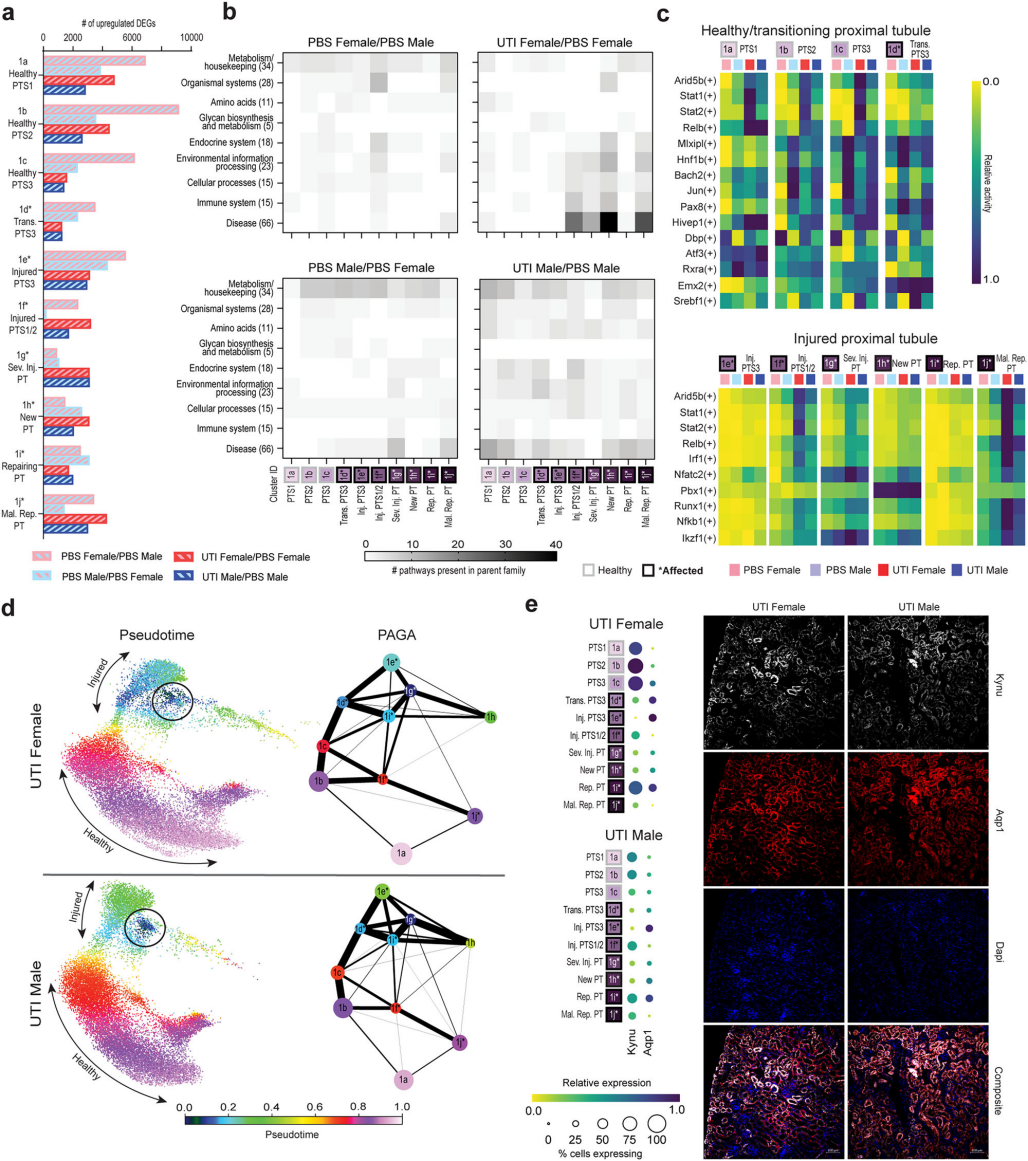
Sex differences in the proximal tubule during UTI. **a** The number of upregulated DEGs (*z*-score > 2.0) in each PT subtype, shown for each pairwise comparison as indicated. **b** Heatmaps depicting the number of KEGG pathways within each parent family that were significantly enriched (FDR < 0.05) in the 200 DEGs most highly upregulated in the first condition compared to the second condition. **c** Activity for the most differentially active transcription factor regulons in each PT cluster during UTI, normalized by row. The upper and lower heatmaps show healthy/transitioning and injured PT cell types, respectively. For each row, the superset of the five most differentially active regulons during UTI in each cell type are plotted. **d** Pseudotime (left) and PAGA (right) analysis of the proximal tubule during UTI. Pseudotime trajectories were rooted at the severely injured cluster (black circles), with light purple representing cells that are most different from the root. For PAGA analysis, the line weights represent connectivity between cell types. **e** Relative expression of *Kynu* and *Aqp1* in the PT of UTI female and male mice (left), with corresponding IF microscopy images (right) of Kynu (white) colocalization with Aqp1^+^ PT cells (red), along with Dapi nuclear staining (blue); scale bars, 100 μm. PT, proximal tubule; S, segment; Trans., transitioning; Inj., injured; Sev. Inj., severely injured; Rep., repairing; Mal. Rep., maladaptive repair.

When sex differences between PBS conditions were examined, females had more upregulated DEGs than males in all PT clusters other than severely injured, new and repairing PT segments (Fig. 5a). Further, PBS females (compared to PBS males) exhibited the most upregulated DEGs among all conditions in all healthy PT segments, along with transitioning and injured PTS3 cells (clusters 1a-1e). However, these upregulated DEGs in PBS females comprised low involvement in any KEGG pathway family; the most activity was observed in metabolism and housekeeping, albeit to a lesser degree than in PBS males (Fig. 5b, left panels; specific pathways in Supplementary Data 6^26^). In the pySCENIC analysis, PBS males exhibited increased TF regulon activity in most cell clusters, compared to PBS females (Fig. 5c and Supplementary Data 7^26^). This was particularly evident in healthy clusters, for example in the *Jun* regulon, well-studied in the immediate-early response process^41^. *Jun* activation in PBS males (reflecting activity at baseline or in response to sterile inoculation) was higher than that of UTI females in most healthy and transitioning PT clusters, consistent with a predisposition to injury upon perturbation of the male kidney.

We next compared PT responses in UTI males or females to PBS mice of the same sex. Unlike in the CD, UTI females exhibited more upregulated DEGs than males in many PT subtypes (Fig. 5a). However, as was seen in the CD, the PT in females still evidenced a more cell-type-restricted response to UTI. Specifically, many injured or regenerating cell clusters (clusters 1f-h and 1j) showed significant upregulated DEGs involved in KEGG pathways relating to environmental information processing, cellular processes, immune system and disease, while pathways from these same families were comparatively downregulated during UTI (i.e., comparatively upregulated in PBS females) in other PT clusters (1a-e and 1i; Fig. 5b, upper right panel, with specific pathways in Supplementary Data 6^26^). Further, considering the most active TF regulons in UTI based on pySCENIC analysis (regardless of sex), UTI females showed the most activity in injured PT clusters, with the most significant regulon activity in injured PTS1/2, severely injured PT, and maladaptive repair clusters (1 f, g, j; Fig. 5c and Supplementary Data 7^26^). TF regulons downregulated in PT during UTI (compared to PBS) are shown in Supplementary Fig. 5a.

In males with UTI (compared to PBS males), the most upregulated DEGs represented more modest enrichment of KEGG pathways across the range of PT cell types, indicating (as seen in the CD) a broader, less cell-type-specific response to UTI than in females (Fig. 5b; specific pathways in Supplementary Data 6^26^). DEGs involved in KEGG pathways reflecting disease were more expressed across virtually all PT clusters during male UTI, in contrast to the cell-type restriction in females. Interestingly, DEGs in disease-related KEGG pathways were comparatively downregulated in healthy PT and injured PTS3 segments in UTI females compared to PBS females (Supplementary Fig. 5b). This effect was not seen in the same comparison in males, indicating that healthy PT cells in females may reduce this type of gene expression during UTI.

As we did in the CD, we performed separate pseudotime analyses of male and female PT clusters during UTI, rooting these analyses at severely injured PT (cluster 1 g; Fig. 5d, circled) to examine the relationships between injured cells and healthy or repairing clusters. Healthy PT cell clusters in male UTI (Fig. 5c, red/purple) demonstrated a more constrained trajectory and darker coloration than those in UTI females, indicating that healthy PT cells more closely resemble severely injured cells in UTI males. While the pseudotime trajectory showed injured PT clusters (Fig. 5d, blue/green) in UTI males as less related to severely injured cells than in UTI females, PAGA plots (a graphical abstraction that reconciles cell clustering with the trajectory inference^42^) showed closer relationships (denoted by thicker connecting lines) among injured clusters in infected males, as well as more connections between healthy and injured clusters (Fig. 5d, right). This abundance of relatedness between healthy and injured PT clusters may indicate that male sex primes the PT for an injurious response to UTI.

Finally, we examined the expression of kynureninase (*Kynu*) in the PT during UTI. The kynurenine pathway is known to reflect early kidney injury and is induced by cytokines such as IFNγ and TNFα^43^, which are produced at higher levels in UTI females at early time points^6,27^. Interestingly, while activation of the kynurenine pathway reduces neutrophil migration and promotes UPEC survival during UTI^44^, inhibition of this pathway promotes renal fibrosis in AKI by enhancing epithelial-to-mesenchymal transition^45^. Our results demonstrated increased *Kynu* expression in UTI females compared to UTI males, especially in healthy and repairing PT clusters (Fig. 5e). UTI males, in contrast, failed to increase *Kynu* expression in injured PT (compared with healthy PT clusters). Immunofluorescence microscopy of PT in UTI females reflected regions of both high and low Kynu positivity, while UTI males showed more diffuse staining and increased colocalization with Aqp1 throughout the PT (Fig. 5e). Further, examination of the kynurenine aminotransferases (KATs) involved in synthesizing kynurenic acid, an endogenous inhibitor of the kynurenine pathway^43^, revealed that UTI females had higher expression of KAT II (*Aadat*) and III (*Kyat3*) in injured PT clusters, while UTI male mice had expression of KAT I (*Kyat1*) and II throughout the PT, with lower expression of KAT II and III compared to females (Supplementary Fig. 5c). In UTI males, expression of these KAT genes mirrored that of *Kynu*, indicating that these cells may be actively inhibiting this pathway. These findings are consistent with a model in which early high-level expression of *Kynu* in PT in females (as opposed to lower, continuous expression in males) acts to better promote repair of UTI-induced injury.

### Sex differences in cell-cell communication during UTI

**Fig. 6:**
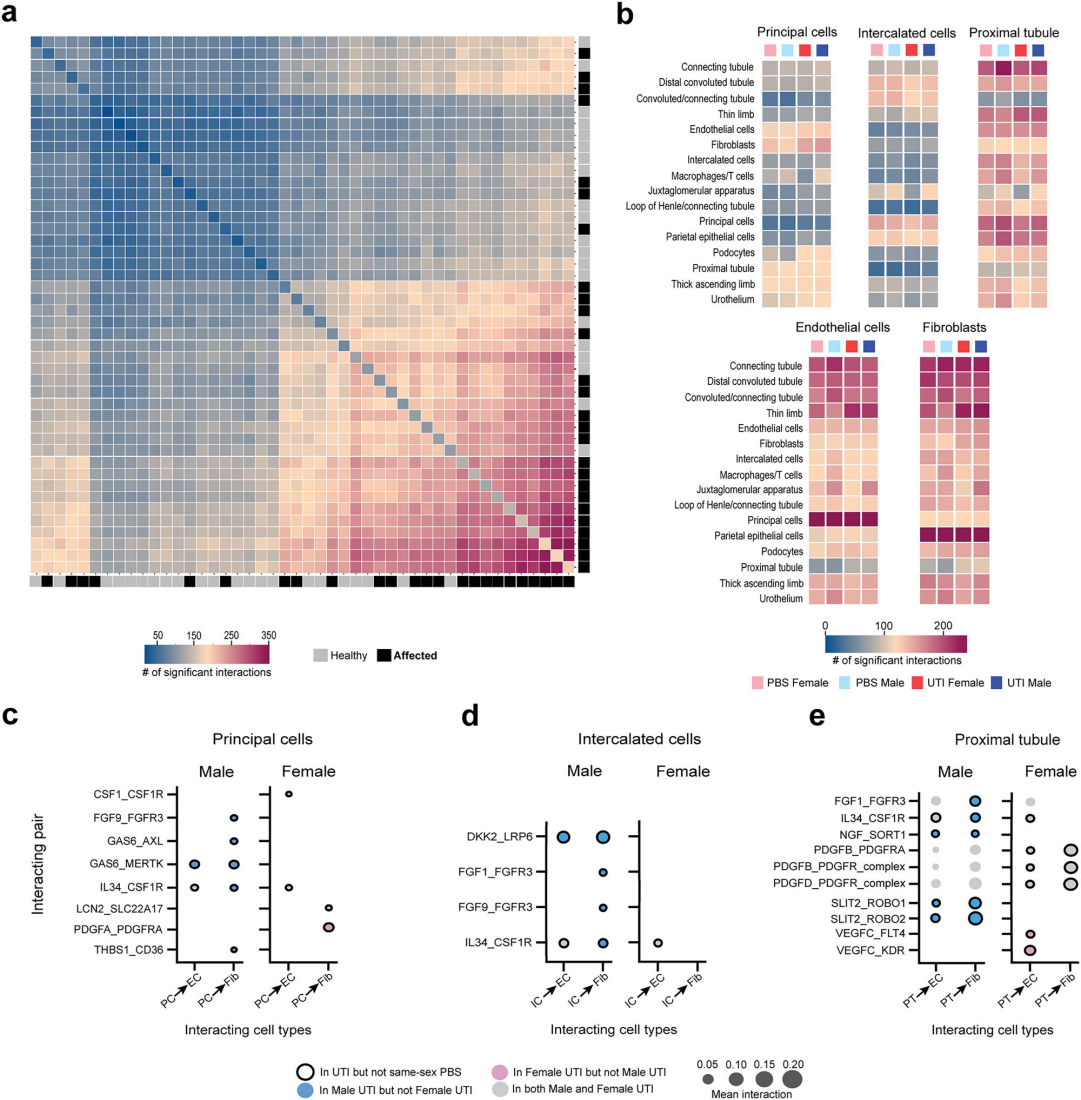
Sex differences in cell-cell communication during UTI. **a** Heatmap of the number of significant cell-cell interactions based on CellPhoneDB analysis, demonstrating that healthy clusters (gray boxes along right and lower borders) exhibit fewer interactions than injured clusters (black boxes along right and lower borders). **b** Heatmaps of the number of significant cell-cell interactions with ligands produced by principal cells, intercalated cells, proximal tubule cells, endothelial cells, and fibroblasts to receptors expressed on other kidney cell types. Cell-cell interactions of ligands secreted by principal cells (**c**), intercalated cells (**d**), and proximal tubule cells (**e**) to receptors on endothelial cells (EC) or fibroblasts (Fib) that were unique to males or females during UTI. Mean interaction indicates the average expression of the ligand/receptor interaction in the corresponding cell types.

At 5 dpi, UPEC are known to interact directly with the CD^7,37^, but we found injury signals throughout the nephron (including in PT) during UTI. We further interrogated this finding by analyzing all 46 clusters using CellPhoneDB, which allows inferences regarding cell-cell communication by examining the combined expression of homologous human ligand-receptor pairs^46^. Overall, affected cell clusters were more frequently involved in measurable communication than healthy clusters (Fig. 6a). When examining the number of cell-cell interactions in only the 16 basic kidney cell types (i.e., not subdivided into healthy versus affected clusters), we found that these interactions were more influenced by sex than by UTI status. In other words, the PBS and UTI-exposed mice of each sex displayed the most comparable numbers of cell-cell interactions. Within the CD, ICs showed relatively few significant communications, interacting mostly with nearby cells such as PCs and DCTs (Fig. 6b). PCs exhibited more frequent and broader interactions, including with endothelial cells, fibroblasts, and PT cell types (Fig. 6b), suggesting that PCs may be chiefly responsible for communication from the CD to the rest of the kidney.

We then examined sex differences in specific interactions between ligands secreted by ICs, PCs, or PT cells to surface-expressed receptors on endothelial cells and fibroblasts, as activation of these latter cell types is associated with renal scarring^47,48^. The interaction set was refined to include only interactions featuring a secreted ligand and with at least one instance of being uniquely present in either the male or female UTI condition, and not in the same-sex PBS condition (Fig. 6c–e and Supplementary Data 8^26^). Males exhibited more unique interactions during UTI, and refinement resulted in no interactions by ICs that were specific to UTI females (Fig. 6d). During UTI, males showed unique interactions reflecting pro-fibrotic responses to injury, including mesenchymal stem cell activation through TGF β1 superfamily signaling^49–51^ (GAS6 and THBS1 by PCs; Fig. 6c) and Wnt signaling^52^ (DKK2 by ICs; Fig. 6d), as well as interactions relating to activation (FGF)^53^ and pro-inflammatory signaling (IL34)^54^ by both ICs and PT cells (Fig. 6d, e). Interestingly, PT cells in female mice participated in PDGFB/D_PDGFR interactions only during UTI (Fig. 6e, black outlines), while such interactions were already present in PBS males (Fig. 6e, no outlines). These interactions on fibroblasts play a major role in renal fibrosis and scarring in AKI^55^; their occurrence even in uninfected males further underscores the increased propensity of males toward renal scarring during experimental pyelonephritis^9^. Potentially moderating this pro-fibrotic tendency in males, PT cells in UTI males also had unique interactions with endothelial cells and fibroblasts via SLIT2 and ROBO1/2 (Fig. 6e). These interactions have been shown to modulate actin rearrangement in various cell types and are proposed to exert anti-fibrotic action in AKI^56^.

The unique cell-cell interactions evident in UTI females, rather than reflecting scarring propensity as in males, instead illuminated pathways of productive repair and defense. While UTI female PCs uniquely interacted with fibroblasts through PDGFA (Fig. 6c), PDGFA is thought to be much less involved in fibrosis compared to PDGFB and PDGFD^57^. Further, UTI female PCs highly expressed CSF1, which can induce a cytokine cascade from endothelial cells to promote activation of reparative macrophages^58,59^. UTI female PCs also expressed LCN2, which limits bacterial growth by sequestering iron during UPEC cystitis^60^ (Fig. 6c). Finally, sex differences in PT and EC communication may also affect cell survival. UTI males were found to participate in NGF_SORT1 signaling, which can promote apoptosis^61^, while UTI females more highly evidenced VEGFC_FLT4/KDR signaling, which promotes EC survival and proliferation^62^ (Fig. 6e).

Investigation of interactions in the reverse direction – between ligands secreted by endothelial cells and fibroblasts and receptors on IC, PC, and PT cells – reflected similar trends (Supplementary Fig. 6 and Supplementary Data 8 and 9^26^). UTI females exhibited no unique interactions, and the secreted ligands of endothelial cells and fibroblasts had no interactions with PT in female mice, regardless of UTI exposure. All unique interactions exhibited by endothelial cells and fibroblasts in UTI males are known to modulate cell migration and proliferation^63–66^, which is indicative of maladaptive repair during AKI^10^ and affected cell status in our model (Supplementary Fig. 6). Taken together, these patterns of unique cell-cell communication during UTI further support a model in which males are predisposed to maladaptive repair and scarring, while females are more successful in antibacterial defense and productive repair.

## Discussion

Single-cell (sc) and single-nucleus (sn) RNA sequencing has been used extensively to study the kidney response to various acute and chronic injuries and perturbations^67,68^. We previously reported sex differences in pathogenesis and outcomes in experimental pyelonephritis. Specifically, male mice exhibit significantly higher incidence of chronic, high-titer kidney infection than females; intra-renal macrophage polarization favoring scarring; and neutrophil functional alterations within the kidney^6–8,69^. Many of these phenotypes are recapitulated when females are exposed to testosterone before infection^8,9,69^. In this study, we used snRNAseq to investigate how sex influences the transcriptional response of the kidney during high-titer pyelonephritis, creating a dataset from nuclei isolated from the kidneys of female, male, and androgen-exposed female C3H/HeN mice 5 days following intravesical inoculation with UPEC strain UTI89 or with PBS (control). At this time point, UPEC are thus far known only to directly interact with the collecting duct, particularly with Aqp2^+^ medullary principal cells^37^. Further, murine ascending pyelonephritis does not affect the whole kidney homogeneously, instead resulting in regional foci of infection and abscess formation surrounded by areas of healthy tissue^7^. Specifically, at 5 dpi, UPEC kidney bacterial communities are just beginning to form^7,37^ and abscess formation is limited. These facts have curtailed our ability to use traditional whole-kidney analyses to understand early kidney responses to UTI and their sex specificity.

Androgen-exposed female (Andro) mice have UTI outcome phenotypes approximating those in males^6–9^, suggesting a strong influence of androgen on sex-discrepant outcomes. With our experimental design, we hoped to identify which gene expression differences between male and female mice were attributable to androgen action. However, the transcriptional profiles of both PBS and UTI-exposed Andro mice in this dataset were overwhelmingly dominated by genes and pathways involved in hormone responses, precluding statistically robust interpretation of comparisons to the male and female transcriptomes. Therefore, in this report we excluded the Andro mice from analyses beyond initial cluster identification, limiting our ability to discern which male-female differences are attributable to androgen effects. The gene expression data from Andro mice are posted online along with the male and female mouse data described in this manuscript; additional post-publication analysis may or may not help to separate the effects of UTI from those of exogenous androgen exposure. The Andro mice received testosterone cypionate for only two weeks before initiation of UTI, and this interval may not have allowed establishment of a new baseline in gene expression.

Using the snRNAseq dataset, we were able to successfully identify and distinguish cells affected by UTI (or by PBS inoculation) from cells that remained healthy. We were able to identify affected cells in almost all 16 kidney cell types, indicating that UTI exerts wide-reaching effects across the kidney and beyond those arising from direct UPEC-host cell interaction. Our cell-cell communication analysis revealed that affected clusters exhibited more interactions with other cells than did healthy ones, and when communication between specific cell types was examined, cells of the CD (both PCs and ICs) evidenced fewer cell-cell interactions than the PT overall, regardless of inoculation condition. Further, cluster assignment in the PT in our dataset closely mirrors that of PT injury during AKI^10^. These data indicate that although UPEC might not directly interact with the PT at 5 dpi, these cells are highly sensitive to UTI-related injury and actively participate in communication with multiple other cell types across the kidney.

**Fig. 7:**
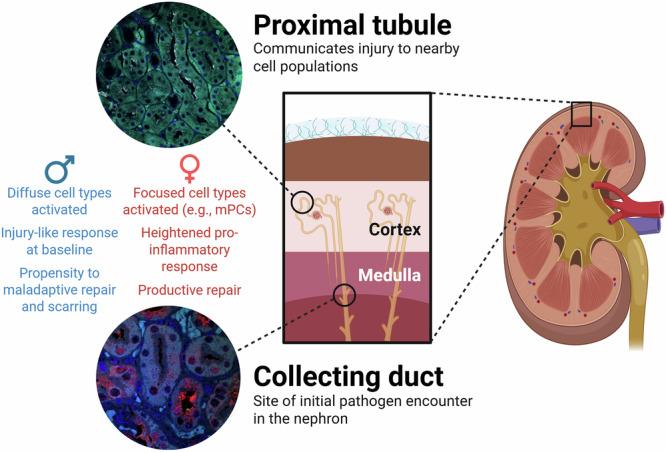
Sex differences in response to UPEC infection of the kidney. Schematic summary of selected conclusions from our analysis. mPC, medullary principal cells. Created in BioRender. https://biorender.com/ae6iqw1.

We chose to focus our investigation on sex differences in the CD – where UPEC are known to directly interact with host cells 5 dpi^37^ – and the PT, where cellular injury and repair pathways have been very well described in noninfectious injury models^10,12^ (Fig. 7). Mice with UTI exhibited increased proportions of injured, severely injured and maladaptive repair PT cells, mimicking the PT injury response reported during non-infectious AKI^10^. In both these nephron segments, males and females had a similar distribution of cells between healthy and affected clusters, but sex had a significant influence on transcriptional responses to UTI. Females exhibited a more targeted response to infection, with few cell types (e.g., mPCs and injured PT clusters) showing marked transcriptional regulation. In contrast, males had a more diffused response to UTI, with both healthy and affected clusters exhibiting upregulation of KEGG pathways and TFs related to disease and pro-inflammatory signaling. Overall, the male response to UTI also was less pronounced (of lower “amplitude”) than that in females (Fig. 7), with UTI females having the highest relative activity of UTI-related TFs in both the CD and PT.

Previous data have shown that in general (through whole-kidney cytokine quantification, Western blotting or flow cytometry), males (or androgenized females) have higher tissue levels of pro-inflammatory cytokines, and increased pro-fibrotic signaling compared to females^6,8,9^. This phenomenon is evidenced in this snRNAseq dataset by significant expression of disease-related KEGG pathways in male mice regardless of inoculation condition. Males in our study also exhibited less defined pseudotime trajectories between clusters in the CD and PT during UTI (i.e., more relatedness between healthy and affected clusters). Further, we examined the expression of *Spp1* in the CD and *Kynu* in the PT. Spp1 is known to induce TGFβ1-dependent myofibroblast activation through increased Smad2/3 phosphorylation^39^, and its expression in UTI females was largely restricted to mPCs, while males exhibited diffuse expression of *Spp1* throughout the CD, consistent with their phenotypic susceptibility to post-pyelonephritic scarring^7,9^. Meanwhile, in the PT, *Kynu* was most expressed in healthy clusters in females, while UTI males again exhibited broad but comparatively lower *Kynu* expression throughout the PT as well as expression of genes involved in *Kynu* pathway inhibition, aligning with reports linking kynurenine pathway inhibition to enhanced epithelial-to-mesenchymal transition in AKI^43,45^. Finally, in other studies examining earlier time points in murine UTI (1 dpi), the innate response in females was earlier and of higher amplitude, while the male response was comparatively dampened and delayed^6,27^.

Interestingly, cells from PBS mice were present in every affected cluster and contributed a similar number of cells to many of these clusters as did UTI-infected mice. The presence of affected cells in PBS mice may reflect responses to VUR inherent to the C3H/HeN strain or be related to the PBS inoculation itself. This ambiguity would be clarified with snRNAseq of kidneys from C3H/HeN mice that are naive (uninoculated) or catheterized (but with no inoculum delivered). Of note, we included only mice in which the inoculation procedure was without difficulty, and there is no known sex difference in the degree of VUR in these mice (e.g., bacterial delivery to the kidney measured 5 min after bladder inoculation is equal between sexes)^70^. In any case, transcriptional profiles in the affected clusters were very different between PBS and UTI. PBS males had increased activity of pro-inflammatory TF regulons in injured CD and PT clusters compared to PBS females, consistent with a propensity toward injury in male mice (Fig. 7).

Though we focused on a single interval representing a “branch point” for sex-discrepant infection outcomes, future single-cell analyses at additional time points (both earlier and later than in the current study) might further illuminate mechanisms underlying sex discrepancy in early immune control of UTI and in outcomes such as renal scarring. Our study details the responses of kidney cell types to UTI, but the standard single-nucleus isolation largely excludes neutrophils, the primary immune responder to bacterial infection in the kidney. As bulk RNA-seq analysis of whole kidney is complicated by the heterogeneous distribution of infection^7^, detailed interrogation of neutrophil transcriptional responses may be best achieved with dedicated single-nucleus approaches designed to capture these cells^71^. Finally, transcriptomic analyses are inherently inferential, and the functional importance of highlighted pathways would be confirmed by additional mechanistic experiments.

In total, the divergence between male and female transcriptional responses to UTI evident in this dataset, at a time point (5 dpi) when these mice exhibit similar bacterial loads and early abscess formation, begins to illuminate the underlying basis for the markedly sex-discrepant outcomes of renal infection in this model^6,7^. The data do not designate a single pathway driving these outcomes; rather, it is clear that male sex exerts broad cellular effects during response to bacterial infection in the kidney, including through testosterone-dependent mechanisms^6,7^.

## Methods

### Bacterial culture

Uropathogenic *E. coli* (UPEC) strain UTI89, originally isolated from a patient with acute cystitis^72^, was grown statically overnight in Luria-Bertani (LB; Becton Dickinson) broth at 37 °C. Cultures were centrifuged at 7500 × *g* at 4 °C before being resuspended to a final density of ~4 × 10^8^ colony-forming units [CFU]/mL in sterile phosphate-buffered saline (PBS).

### Animals

All animal studies were approved in advance by the Washington University Institutional Animal Care and Use Committee (AWA #D16-00245, protocol #24-0037). We have complied with all relevant ethical regulations for animal use. For androgen exposure, 5-week-old female C3H/HeN mice (Envigo #040) were given weekly intramuscular injections of 150 mg/kg testosterone cypionate in cottonseed oil (TC; McKesson Medical) for 2 weeks before induction of urinary tract infection (UTI). In other groups, 5-week-old male or female C3H/HeN mice (Envigo #040) received weekly intramuscular injections of cottonseed oil. All groups of mice underwent experimental UTI at 7 weeks of age via transurethral inoculation (under inhalational anesthesia with 2% isoflurane for ~2 min) with 1–2 × 10^7^ CFU of UTI89 in 50 μL PBS or with sterile PBS^73–75^.

### Determination of bacterial loads

At 5 dpi, mice were sacrificed via CO_2_ asphyxiation (12 L/min for 3 min followed by 3 min dwell), and bladders and kidneys were aseptically removed. Bladders were homogenized in 4 °C PBS to a final volume of 1 mL, and homogenates were serially diluted and plated on LB agar. Individual kidneys were placed in 1.5-mL tubes and flash frozen in liquid nitrogen before storage at −80 °C. As bladder and kidney bacterial loads are correlated^6,8^, high-titer infection in UPEC-infected mice was defined by bladder titers above 10^6^ CFU/bladder.

### Isolation of nuclei

For nuclear preparation^18,19^, flash-frozen kidneys were minced on ice into cold nuclei lysis buffer (Sigma #NUC101) supplemented with RNasin Plus (Fisher #PRN2615) and SUPERaseIN (ThermoFisher #AM2696), then homogenized using a Dounce homogenizer. 2 mL of lysis buffer was added to the homogenate before incubating for 5 min on ice, and the suspension was passed through a 40-µm strainer (Puriselect #43-50040-51) and centrifuged at 500 × *g* for 5 min at 4 °C. The resulting pellet was washed with lysis buffer and incubated for 5 min at 4 °C before being centrifuged again. The pellet was resuspended in nuclei suspension buffer (Dulbecco’s PBS [Sigma], RNasin Plus) and passed through a 5-µm strainer (Puriselect #43-50005-03) before nuclei were counted on a hemacytometer. Nuclei were diluted to a final concentration of 1200 nuclei/µL in 1% bovine serum albumin, 0.2 U/µL RNasin in DPBS and sequenced immediately. The left kidney was used for each sample, except for the repeated PBS-inoculated male samples (see Sequencing section below), where the contralateral right kidney was used.

### Sequencing

A total of 18 samples were sequenced. Library prep, barcoding, and pooling were performed on the 10X Chromium platform. Sequencing was performed on an Illumina NovaSeq 6000 instrument. The sequencing core was unaware of the group assignment of samples. Among the 18 samples, two samples (PBS-inoculated males) were resequenced due to low nuclei counts or out-of-range ambient RNA content on initial sequencing.

### Analysis of single-nucleus RNA sequencing data

Initial analysis of sequencing data was performed using 10X Genomics CellRanger software (version 6.0.1). CellBender^20^ (version 0.1.0) was used to remove ambient RNA signals, using stringent thresholds (CellBender expected number of cells 10,000; CellRanger Total_droplets_included 100%). Quality control, filtering, and downstream analyses were performed using Scanpy^76^ (version 1.9.3). In filtering steps, we required (i) ≥1200 genes/cell (chosen to obtain a realistic number of cells per dataset while minimizing noise; (ii) ≤5% mitochondrial genes; and (iii) that each gene be present in ≥3 cells. Analysis steps included normalization, log transform, identification of highly variable genes, and principal component analysis. Harmony^22^ (version 0.0.6) was used for integrating single-cell data from multiple experiments across both batch and condition. Doublets were removed with Scrublet^21^ (version 0.2.3). After computing a neighbor map, UMAP was used for dimensional reduction, followed by cell clustering using the leiden algorithm^77^. Genes differing between groups of cells were ranked using a t-test (scanpy method t-test_overestim-var). Cirrocumulus^78^ was used for visualization.

### Cell type clustering and annotations

Cells were initially clustered into 98 clusters, using leiden clustering with the parameter “resolution = 5.0”. These 98 clusters were then manually grouped at three different levels of resolution: (i) 16 general cell types; (ii) 30 specific cell types; and (iii) 46 specific cell types, each split into healthy versus affected groups (Supplementary Data 10^26^). In total, 6631 cells were removed from analysis. Cell type annotations were determined by manual inspection of marker genes and comparison to previous works^10–13,30–36^, and validated using integration mapping and automatic transfer of cell type annotations from a previous study of kidney cells^30^, using the label transfer approach in Seurat^79^ (version 4.3.0).

As the cell cluster originally labeled as immune cells (containing 4890 cells) initially appeared to also contain non-immune cells (expressing both PT-specific *Lrp2* and immune cell marker *Ptprc*), it was manually clustered at higher resolution. Using the leiden algorithm in Scanpy with the parameter “resolution = 0.25,” we split these immune cells into five new clusters. Two of these new clusters (*Lrp*^–^, *Ptprc*^+^) were retained and combined into a single immune cell cluster, while the other three clusters (*Lrp*^+^, *Ptprc*^+^; containing a total of 3482 cells) were discarded from analysis, as they did not exclusively represent immune cells. The final number of cells subjected to downstream analysis, across all conditions, was 248,483.

### KEGG pathway enrichment

ShinyGO^80^ (version 0.80) was used to determine significantly enriched KEGG pathways (FDR < 0.05) from the 200 most upregulated DEGs by *z*-score for each of the comparisons described in Results. Parent families were determined by using the KEGG BRITE database^81^. All significantly enriched KEGG pathways for each of the cell types or conditions described in the results were analyzed together, with the figures representing the number of KEGG pathways from any cluster in a given parent family that are present in a specific cluster or condition.

### GO term enrichment

GO enrichment analysis was performed using the Gene Ontology Database^82–84^ from the 200 most upregulated DEGs by *z*-score in a given healthy or affected cluster compared to all other clusters. Parent families were determined using AmiGO^85^. Differences in GO term enrichment in healthy and affected clusters were limited to significantly enriched GO terms (FDR < 0.05) terms that were unique to each cluster type while being present in at least three healthy or affected clusters. GO terms identifying organ-specific processes irrelevant to the kidney were also removed.

### Additional downstream analyses

Pseudotime analysis was performed separately for each experimental condition using Diffusion Pseudotime^38^ in Scanpy, and PAGA was used to generalize relationships between cell types^42^. Regulon analysis was performed using pySCENIC^24^ (version 0.12.1). For pySCENIC, we re-ran the Scanpy pipeline keeping all genes, including rare genes. Using pySCENIC outputs, we performed comparisons between conditions as we did for expression data, using a t-test. Analysis of cell-cell communication was performed separately for each experimental condition using CellPhoneDB^46^ (version 5.0.0). To use CellPhoneDB, mouse cells were mapped onto human cells using pre-computed homologs from MGI^86^ version 6.18.

### Histology and immunofluorescence (IF) microscopy

For histology and IF studies, mice were anesthetized with isoflurane before perfusion with cold, sterile PBS. Kidneys were decapsulated, bisected, and fixed in 4 °C 4% paraformaldehyde in PBS before incubation in 30% sucrose overnight at 4 °C. Fixed kidneys were then mounted in OCT (Tissue-tek) before cryosectioning into 5–8 µm sections and mounting on Superfrost microscope slides.

Histology sections were rinsed with PBS to remove the OCT, then washed with deionized water before staining with Hematoxylin Solution, Gill No. 2 (Sigma #GHS232). Slides were washed again, treated with Bluing Reagent (Epredia #6769001) before being washed and stained with Eosin Y (Sigma #HT110216). Slides were then dehydrated with ethanol and cleared with xylenes before being mounted with Poly-Mount Xylene (Polysciences #24176) and imaged on an Olympus BX40 light microscope (Tokyo, Japan) with an attached Axiocam 305 color camera (Zeiss, Oberkochen, Germany).

For IF microscopy, rinsed slides were permeabilized with 0.25% Triton X-100 (Sigma) in PBS for 10 min, then blocked for 1 h with 10% fetal bovine serum (Gibco) in PBS before staining with primary antibodies against aquaporin-2 (Aqp2-JaneliaFluor 646 for PCs; Novus Biologicals #NB110-74682JF646, 1:200), V-type protein ATPase subunit B (Atp6v1b-JaneliaFluor 646 for ICs; Novus Biologicals #NBP2-70237JF646, 1:200), aquaporin-1 (Aqp1 for PT; Santa Cruz Biotechnology #sc-25287, 1:50), secreted phosphoprotein 1 (Spp1-JaneliaFluor 525; Novus Biologicals #AF808JF525, 1:100), or kynureninase (Kynu; ThermoFisher #PA593121, 1:100) in blocking buffer. Slides were washed with PBS; for Aqp1 and Kynu-stained slides, secondary staining with goat anti-mouse-AlexaFluor488 (Molecular Probes #A-21121, 1:200) and donkey anti-rabbit-AlexaFluor 594 (Jax Immuno #711-585-152, 1:200) was performed, followed by an additional PBS wash. Slides were stained with 4’,6-diamidino-2-phenylindole (DAPI, 1:5000) in PBS for 5 min, washed and mounted with Prolong Gold Antifade Reagent (ThermoFisher Scientific #P36930). Stained slides were imaged with a Zeiss LSM 880 Airyscan confocal microscope.

### Statistics and reproducibility

Bladder bacterial loads were compared using a Kruskal-Wallis test. Statistical parameters used throughout the RNAseq data analyses are indicated in the respective Methods sections above.

### Reporting summary

Further information on research design is available in the Nature Portfolio Reporting Summary linked to this article.

## Supplementary information

Supplementary Information

Reporting Summary

## Data Availability

Gene expression data have been submitted to the Gene Expression Omnibus (GEO) under accession number GSE296327, and Illumina reads have been submitted to NCBI’s Short Read Archive (SRA) under NCBI bioproject PRJNA1182331. Additional source data behind the figures in the paper are available online^26^ as Supplementary Data 1–10. Any remaining information can be obtained from the corresponding authors upon reasonable request.
