# Supplementary material for: Single-nucleus transcriptomics illuminates sex differences during murine Escherichia coli pyelonephritis
Source: Commun Biol. 2026 Mar 31;9:711. doi: 10.1038/s42003-026-09946-8 (PMC13201655; doi:10.1038/s42003-026-09946-8)
Supplement: Supplementary file 1 — Supplementary Information [file 42003_2026_9946_MOESM1_ESM.pdf]

Supplementary Information

Single-nucleus transcriptomics illuminates sex differences during murine *Escherichia coli* pyelonephritis

Teri N. Hreha,[1]* Abigail L. Manson,[4]* Christina A. Collins,[1] Haojia Wu,[2] Christophe Georgescu,[5] Benjamin D. Humphreys,[2] Ashlee M. Earl,[4†] and David A. Hunstad[1,3†]

[1]Department of Pediatrics, Washington University School of Medicine, St. Louis, MO, USA

[2]Department of Medicine, Washington University School of Medicine, St. Louis, MO, USA

[3]Department of Molecular Microbiology, Washington University School of Medicine, St. Louis, MO, USA

[4]Infectious Disease and Microbiome Program, Broad Institute of MIT and Harvard, Cambridge, MA, USA

[5]Genomics Platform, Broad Institute of MIT and Harvard, Cambridge, MA, USA

*These authors contributed equally.

†These authors jointly supervised this work.

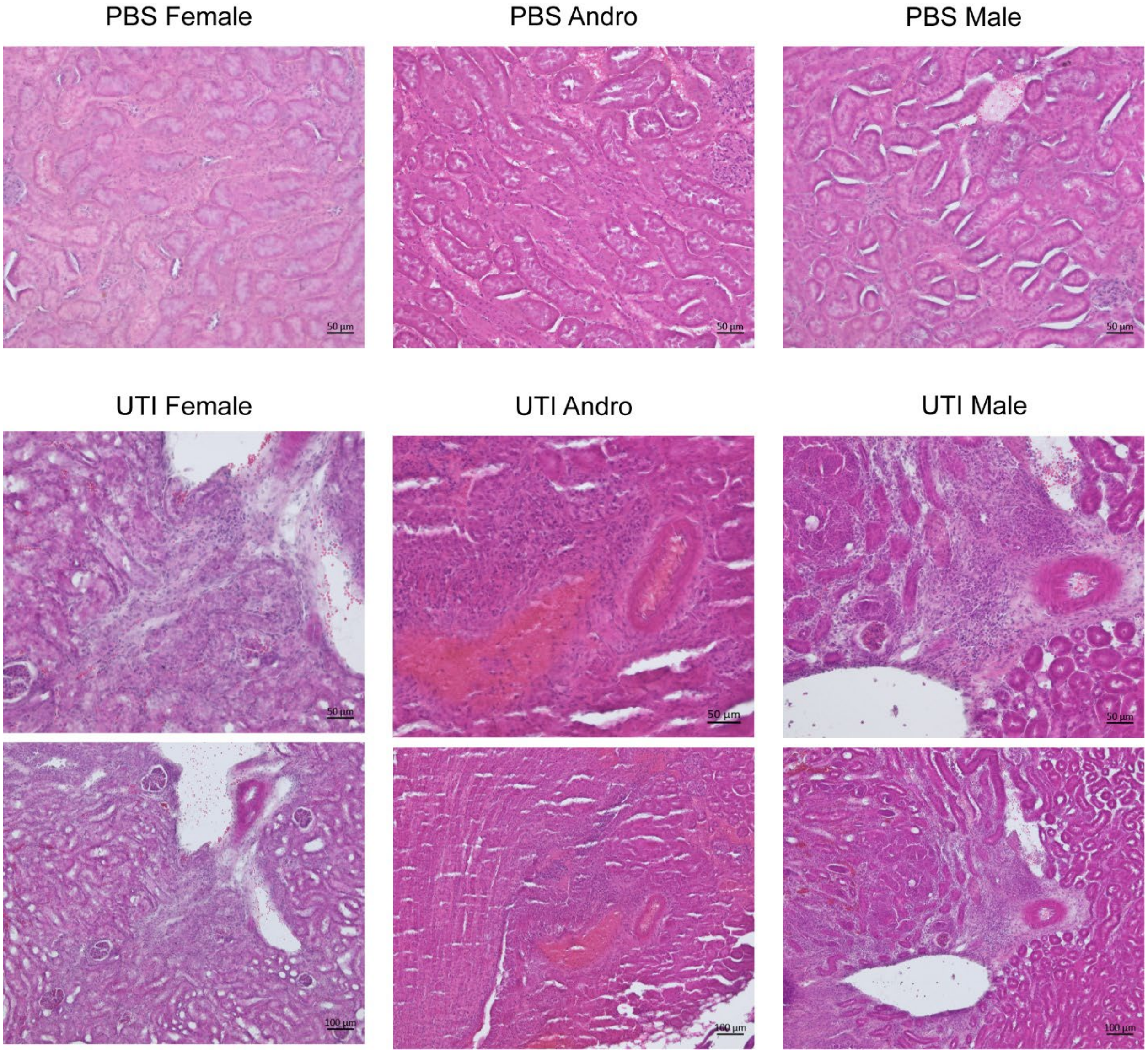

Supplementary Figure 1. Example hematoxylin and eosin-stained kidney sections from female, Andro, and male mice exposed to PBS (top) and UTI (bottom), 5 days post inoculation.

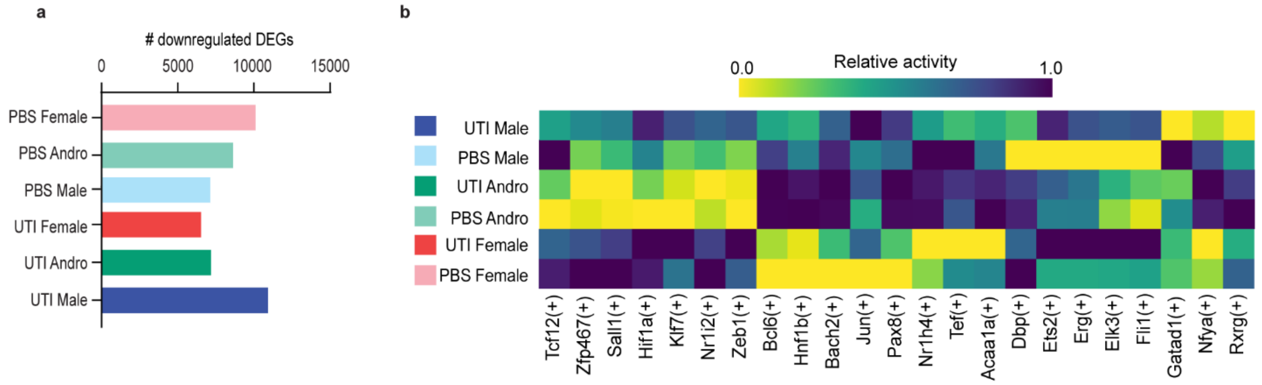

Supplementary Figure 2. a, The number of downregulated DEGs (z-score < -2.0) in each condition compared to all other conditions. **b,** Relative activity for the most downregulated transcription factor regulons in each condition, as compared to all other conditions. For each grouping, the superset of the five most downregulated regulons in each cell type are plotted.

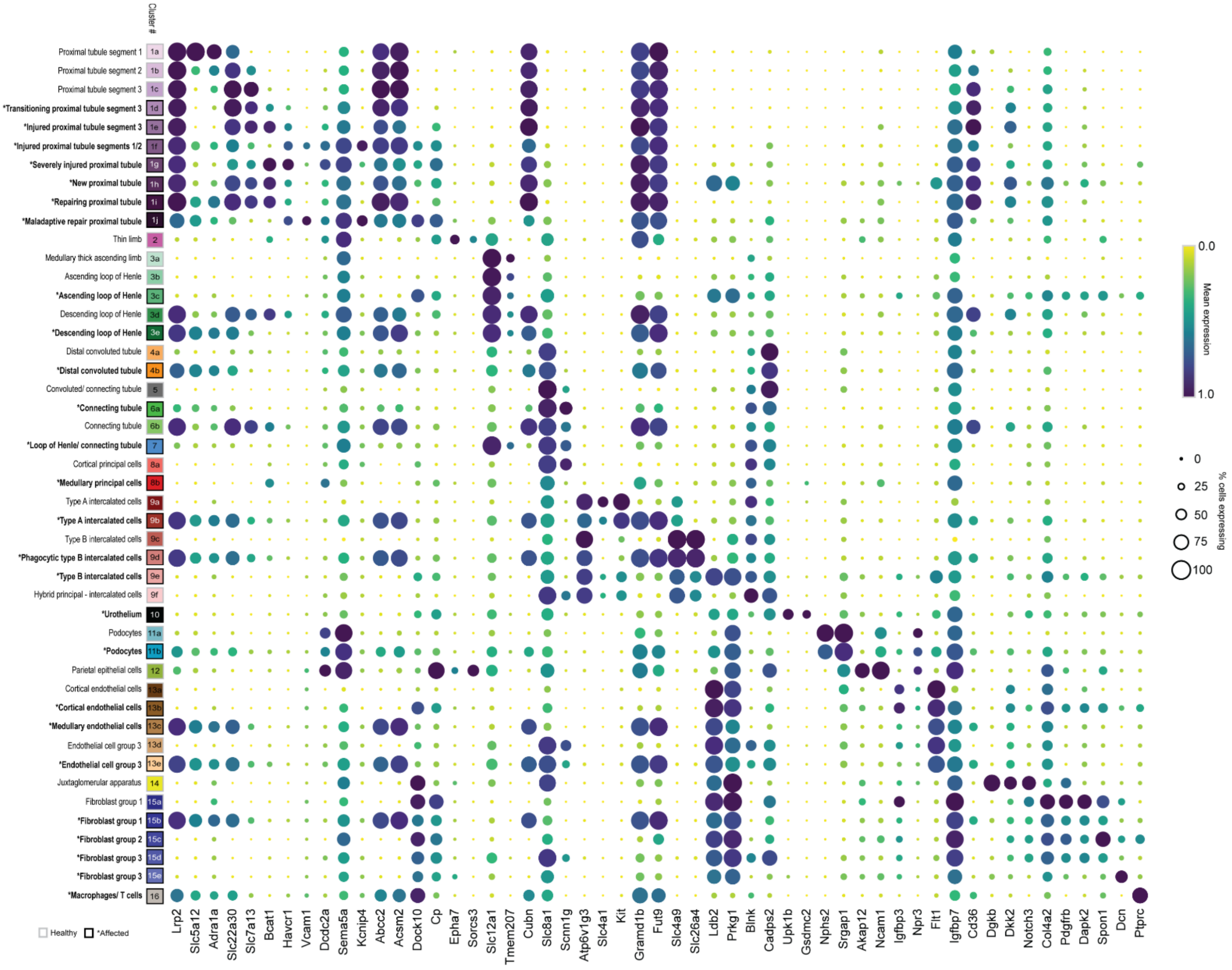

Supplementary Figure 3. Marker genes for the set of 46 cell type clusters, containing healthy clusters (outlined in light grey boxes) and affected clusters (bolded and outlined in black boxes). Color indicates mean expression, and circle size represents the percent of cells expressing this gene.

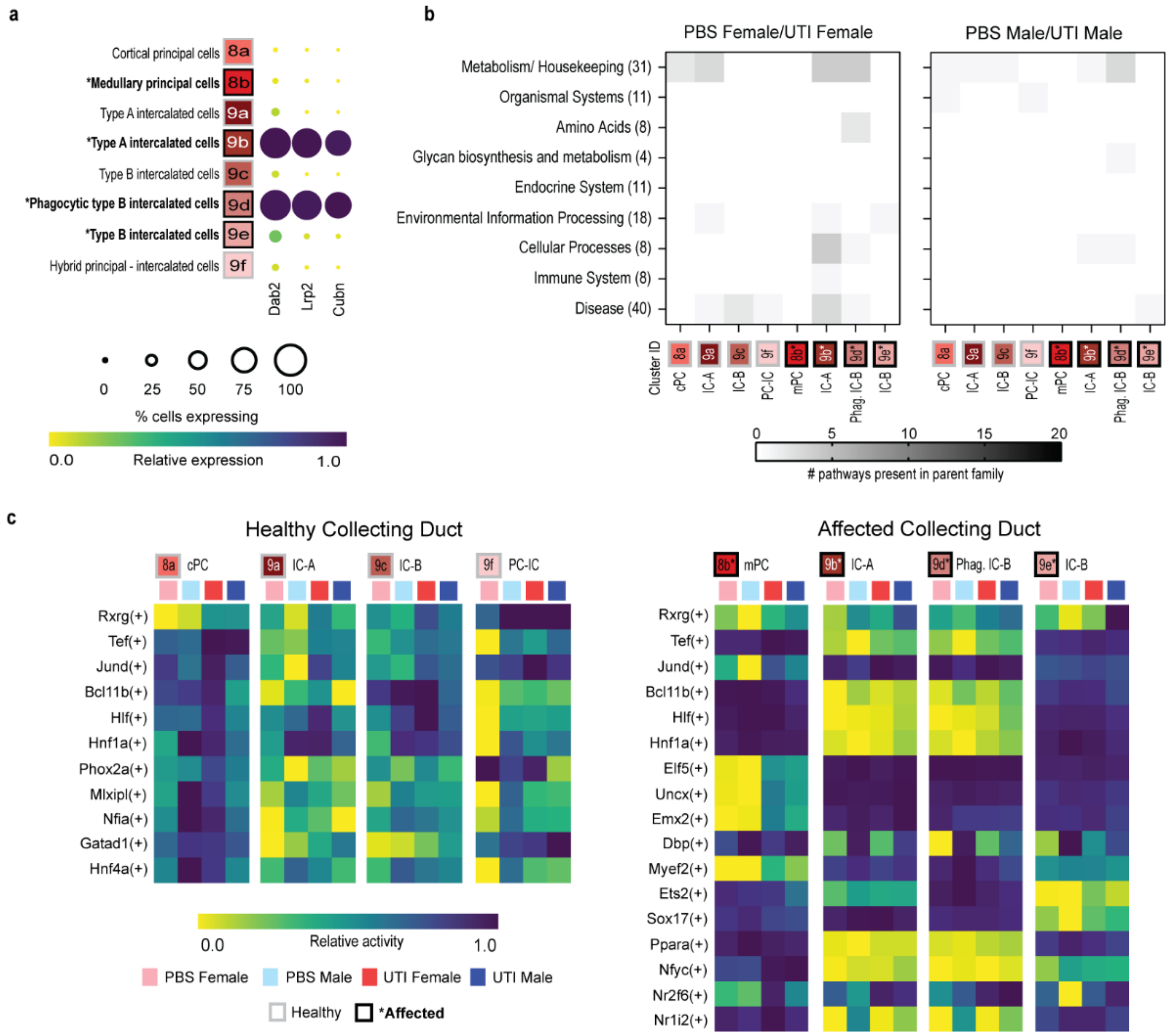

Supplementary Figure 4. a, Genes involved in phagocytosis are expressed in both affected Type A and a subset of affected Type B intercalated cells. Color indicates mean expression, and circle size represents the percent of cells expressing each gene. **b,** Heatmaps depicting the number of KEGG pathways within each parent family that were significantly enriched (FDR < 0.05) within the 200 most upregulated DEGs when comparing PBS mice to their UTI counterparts. **c,** Relative activity for the most downregulated transcription factor regulons in the collecting duct during UTI, as compared to PBS conditions. The left and right heatmaps show grouped healthy and affected cell types, respectively. For each grouping, the superset of the five most downregulated regulons in each cell type are plotted. Healthy clusters are outlined in light grey boxes, and affected clusters are bolded and outlined in black boxes.

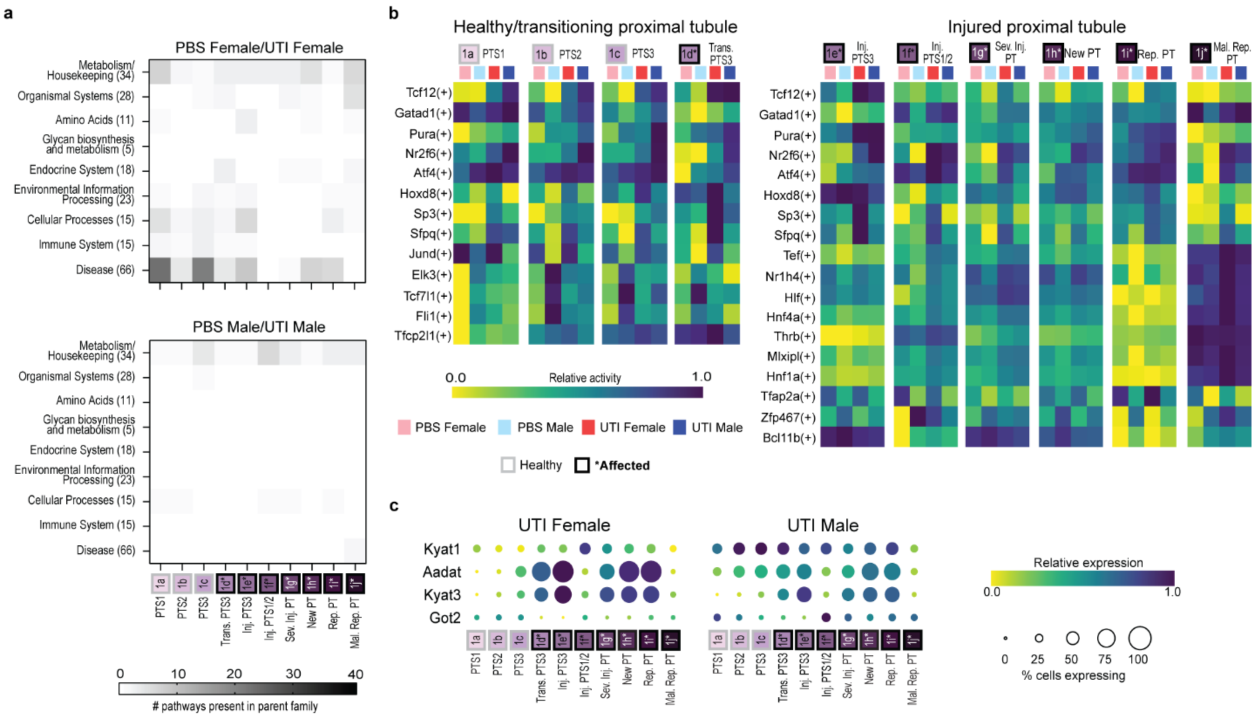

Supplementary Figure 5. a, Heatmaps depicting the number of KEGG pathways within each parent family that were significantly enriched (FDR < 0.05) within the 200 most upregulated DEGs when comparing PBS mice to their UTI counterparts. **b,** Relative activity for the most downregulated transcription factor regulons in the proximal tubule during UTI, as compared to PBS conditions. The left and right heatmaps show grouped healthy and affected cell types, respectively. For each grouping, the superset of the five most downregulated regulons in each cell type are plotted. Healthy clusters are outlined in light grey boxes, and affected clusters are bolded and outlined in black boxes. **c,** Gene expression plots of kynurenine aminotransferases I (*Kyat1*), II (*Aadat*), III (*Kyat3*) and IV (*Got2*) in the proximal tubule of UTI female and male mice.

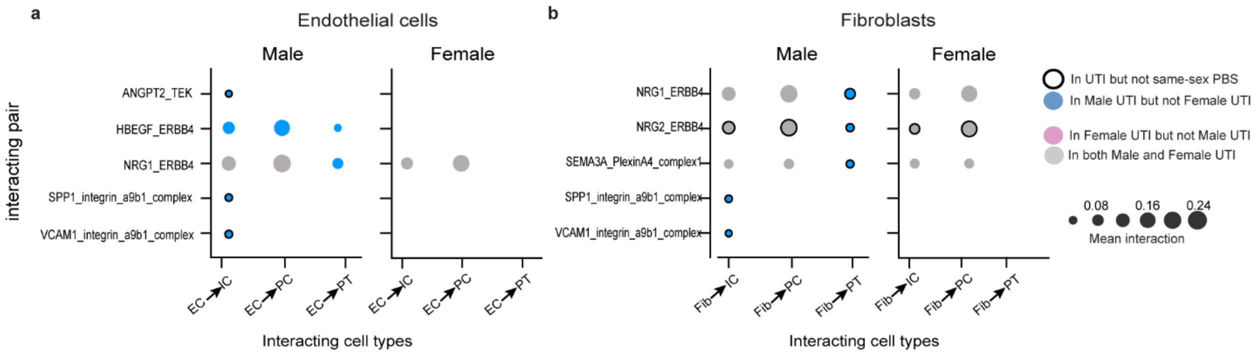

Supplementary Figure 6. Cell-cell interactions of ligands secreted by endothelial cells (**a**) and fibroblasts (**b**) to receptors on intercalated cells (IC), principal cells (PC), or proximal tubule cells (PT) that were unique to males or females during UTI.

Supplementary Data provided online **doi: 10.6084/m9.figshare.31411557**

Supplementary Data 1. Differences between conditions in pySCENIC data in Male, Female, and Andro mice.

Supplementary Data 2. KEGG pathways significantly enriched in the 200 most upregulated DEGs in Male, Female, and Andro mice.

Supplementary Data 3. GO pathways significantly enriched in the 200 most upregulated DEGs in each cluster, refined to pathways present in at least 3 healthy or affected clusters.

Supplementary Data 4. KEGG pathways significantly enriched in the 200 most upregulated DEGs comparing UTI and PBS conditions in the same sex, or between male and female PBS-inoculated mice, in the collecting duct.

Supplementary Data 5. Differences between pySCENIC data in the collecting duct, comparing UTI versus PBS conditions, along with activity of the most and least active TF regulons in UTI by condition.

Supplementary Data 6. KEGG pathways significantly enriched in the 200 most upregulated DEGs comparing UTI and PBS conditions in the same sex, or between male and female PBS-inoculated mice, in the proximal tubule.

Supplementary Data 7. Differences between pySCENIC data in the proximal tubule, comparing UTI versus PBS conditions, along with activity of the most and least active TF regulons in UTI by condition.

Supplementary Data 8. CellPhone DB output by condition, from cell type clustering with both 46 and 16 clusters.

Supplementary Data 9. Condition-specific principal cell, intercalated cell, and proximal tubule CellPhoneDB interactions.

Supplementary Data 10. Cell cluster identification and gene expression tables for 98-cluster Leiden and 16- and 46-cluster analyses.
