# Supplementary material for: Single-nucleus transcriptomics illuminates sex differences during murine Escherichia coli pyelonephritis
Source: Commun Biol. 2026 Mar 31;9:711. doi: 10.1038/s42003-026-09946-8 (PMC13201655; doi:10.1038/s42003-026-09946-8)
Supplement: Supplementary file 2 — Reporting Summary [file 42003_2026_9946_MOESM2_ESM.pdf]

Corresponding author(s): David A. Hunstad, Ashlee M. Earl

Last updated by author(s): Feb 25, 2026

Reporting Summary

Nature Portfolio wishes to improve the reproducibility of the work that we publish. This form provides structure for consistency and transparency in reporting. For further information on Nature Portfolio policies, see our Editorial Policies and the Editorial Policy Checklist.

Statistics

For all statistical analyses, confirm that the following items are present in the figure legend, table legend, main text, or Methods section.

n/a	Confirmed	
☐	☒	The exact sample size (*n*) for each experimental group/condition, given as a discrete number and unit of measurement
☐	☒	A statement on whether measurements were taken from distinct samples or whether the same sample was measured repeatedly
☐	☒	The statistical test(s) used AND whether they are one- or two-sided<br>*Only common tests should be described solely by name; describe more complex techniques in the Methods section.*
☐	☒	A description of all covariates tested
☐	☒	A description of any assumptions or corrections, such as tests of normality and adjustment for multiple comparisons
☐	☒	A full description of the statistical parameters including central tendency (e.g. means) or other basic estimates (e.g. regression coefficient) AND variation (e.g. standard deviation) or associated estimates of uncertainty (e.g. confidence intervals)
☐	☒	For null hypothesis testing, the test statistic (e.g. *F*, *t*, *r*) with confidence intervals, effect sizes, degrees of freedom and *P* value noted<br>*Give P values as exact values whenever suitable.*
☒	☐	For Bayesian analysis, information on the choice of priors and Markov chain Monte Carlo settings
☒	☐	For hierarchical and complex designs, identification of the appropriate level for tests and full reporting of outcomes
☒	☐	Estimates of effect sizes (e.g. Cohen's *d*, Pearson's *r*), indicating how they were calculated

Our web collection on statistics for biologists contains articles on many of the points above.

Software and code

Policy information about availability of computer code

Data collection	Library prep, barcoding, and pooling were performed on the 10X Chromium platform. Sequencing was performed on an Illumina NovaSeq 6000 instrument.
Data analysis	Initial analysis of sequencing data was performed using 10X Genomics CellRanger software (version 6.0.1). CellBender16 (version 0.1.0) was used to remove ambient RNA signals, using stringent thresholds. Quality control, filtering, and downstream analyses were performed using Scanpy (version 1.9.3). Harmony (version 0.0.6) was used for integrating single-cell data from multiple experiments across both batch and condition. Doublets were removed with Scrublet (version 0.2.3). After computing a neighbor map, UMAP was used for dimensional reduction, followed by cell clustering using the leiden algorithm. Cirrocumulus was used for visualization. Pseudotime analysis was performed separately for each experimental condition using Diffusion Pseudotime in Scanpy, and PAGA was used to generalize relationships between cell types. Regulon analysis was performed using pySCENIC20 (version 0.12.1). Analysis of cell-cell communication was performed separately for each experimental condition using CellPhoneDB (version 5.0.0). To use CellPhoneDB, mouse cells were mapped onto human cells using pre-computed homologs from MGI version 6.18.

For manuscripts utilizing custom algorithms or software that are central to the research but not yet described in published literature, software must be made available to editors and reviewers. We strongly encourage code deposition in a community repository (e.g. GitHub). See the Nature Portfolio guidelines for submitting code & software for further information.

Data

Policy information about availability of data

All manuscripts must include a data availability statement. This statement should provide the following information, where applicable:

- Accession codes, unique identifiers, or web links for publicly available datasets
- A description of any restrictions on data availability
- For clinical datasets or third party data, please ensure that the statement adheres to our policy

Gene expression data have been submitted to the Gene Expression Omnibus (GEO) under accession number GSE296327, and Illumina reads have been submitted to NCBI's Short Read Archive (SRA) under NCBI bioproject PRJNA1182331. Supplementary Data 1-10 are posted at Figshare.

Research involving human participants, their data, or biological material

Policy information about studies with human participants or human data. See also policy information about sex, gender (identity/presentation), and sexual orientation and race, ethnicity and racism.

Reporting on sex and gender	No human participants or data were involved.
Reporting on race, ethnicity, or other socially relevant groupings	No human participants or data were involved.
Population characteristics	No human participants or data were involved.
Recruitment	No human participants or data were involved.
Ethics oversight	No human participants or data were involved.

Note that full information on the approval of the study protocol must also be provided in the manuscript.

Field-specific reporting

Please select the one below that is the best fit for your research. If you are not sure, read the appropriate sections before making your selection.

☒ Life sciences ☐ Behavioural & social sciences ☐ Ecological, evolutionary & environmental sciences

For a reference copy of the document with all sections, see nature.com/documents/nr-reporting-summary-flat.pdf

Life sciences study design

All studies must disclose on these points even when the disclosure is negative.

Sample size	Three mice from each UTI group (Female, Andro, Male) were selected for their high and comparable bladder bacterial loads and visible renal abscesses at the time of sacrifice; three mice from each control (PBS) group were confirmed to have sterile bladders at the time of sacrifice.
Data exclusions	As detailed in the manuscript, domination of transcriptional responses in Andro mice by hormone-processing pathways permeated through our downstream analyses, precluding reliable assignment of the effects of UTI in Andro mice. As a result, we included the Andro groups in all cell-clustering determinations, but our subsequent analyses focused only on the female and male groups. All data from all groups are being made available as described in the Data Availability section.
Replication	Due to the extensive technical and analytical processes required to generate the single-cell RNAseq dataset, we corroborated selected findings with immunofluorescence microscopy.
Randomization	All mice were inbred and phenotypically comparable.
Blinding	Staff in the WashU core facility that performed library prep, sequencing, initial QC, and resequencing were not aware of the experimental conditions for each sample.

Reporting for specific materials, systems and methods

We require information from authors about some types of materials, experimental systems and methods used in many studies. Here, indicate whether each material, system or method listed is relevant to your study. If you are not sure if a list item applies to your research, read the appropriate section before selecting a response.

Materials & experimental systems

n/a	Involved in the study	
☐	☒	Antibodies
☒	☐	Eukaryotic cell lines
☒	☐	Palaeontology and archaeology
☐	☒	Animals and other organisms
☒	☐	Clinical data
☒	☐	Dual use research of concern
☒	☐	Plants

Methods

n/a	Involved in the study	
☒	☐	ChIP-seq
☒	☐	Flow cytometry
☒	☐	MRI-based neuroimaging

Antibodies

Antibodies used	For immunofluorescence microscopy, primary antibodies included: aquaporin-2 (Aqp2-AlexaFluor 488 for PCs; Santa Cruz Biotechnology #sc-515770), V-type protein ATPase subunit B (Atp6v1b-AlexaFluor 488 for ICs; Novus Biologicals #NBP2-70237AF488), aquaporin-1 (Aqp1 for PT; Santa Cruz Biotechnology #sc-25287), secreted phosphoprotein 1 (Spp1-MFluor Violet 610; Novus Biologicals #NBP-20774MFV610), and kynureninase (Kynu; ThermoFisher #PA593121). Secondary antibodies included goat anti-mouse-AlexaFluor488 (Molecular probes #A-21121) and donkey anti-rabbit-AlexaFluor 594 (Jax Immuno #711-585-152).
Validation	Specificity and suitability of antibodies for IF was per the manufacturers' websites. We tested several concentrations of each antibody on mouse kidney sections to optimize conditions and exclude background reactivity.

Animals and other research organisms

Policy information about studies involving animals; ARRIVE guidelines recommended for reporting animal research, and Sex and Gender in Research

Laboratory animals	C3H/HeN mice (Envigo #040) infected at 7 weeks of age
Wild animals	No wild animals were involved.
Reporting on sex	Both mouse sexes were used. Sex differences are the basis of most of the analyses reported in the paper.
Field-collected samples	No field-collected samples were involved.
Ethics oversight	All animal studies were approved in advance (protocol #24-0037) by the Institutional Animal Care and Use Committee (IACUC) at Washington University (AWA #D16-00245), which is fully accredited by the Association for Assessment and Accreditation of Laboratory Animal Care (AAALAC International).

Note that full information on the approval of the study protocol must also be provided in the manuscript.

Plants

Seed stocks	No plants were involved in the study.
Novel plant genotypes	No plants were involved in the study.
Authentication	No plants were involved in the study.
